# Association between ERCC5 gene rs17655 polymorphism and the risk of breast cancer: an updated meta-analysis

**DOI:** 10.1265/ehpm.24-00385

**Published:** 2026-07-01

**Authors:** Wen-Shu Luo, Anqi Shi, Zheng Wang, Shu-Zhen Zheng, Junhong Li

**Affiliations:** 1 Department of Chronic- Non- communicable Disease Prevention and Control, Changzhou Center for Disease Control and Prevention, Changzhou Advanced Institute of Public Health, Nanjing Medical University, Changzhou, 213022, Jiangsu, People’s Republic of China; 2 Kangda College of Nanjing Medical University, Lianyungang, 222000, Jiangsu, People’s Republic of China; 3 Department of Organization and Human Resources, Changzhou Center for Disease Control and Prevention, Changzhou Advanced Institute of Public Health, Nanjing Medical University, Changzhou, 213022, Jiangsu, People’s Republic of China; 4 Department of Chronic Non communicable Disease Prevention and Control, Xinbei District Center for Disease Control and Prevention, Xinbei District, Changzhou, 213022, Jiangsu, People’s Republic of China; 5 Department of Physical and Chemical Laboratory, Changzhou Center for Disease Control and Prevention, Changzhou, 213022, Jiangsu, People’s Republic of China

**Keywords:** Breast cancer, ERCC5, Genetics, Meta-analysis, Polymorphism

## Abstract

**Background:**

Previously, several studies have focused on the correlation between SNPs of the excision repair cross-complementation group 5 (*ERCC5*) gene and breast cancer susceptibility, and the rs17655 (*Asp1104His*) has been mostly studied among many SNPs of *ERCC5* gene. However, contradictory results also existed among these published articles. So, we performed this meta-analysis to evaluate the relationship between the rs17655 of *ERCC5* gene and the susceptibility to breast cancer.

**Methods:**

All analyses in this meta-analysis were performed using Stata 15.0 version software. The heterogeneity test was performed using the chi-square-based Q-test and the I-square test. Sensitivity analysis was performed to estimate the influence of the combined ORs caused by individual data. Begg’s funnel plot method was used for the publication bias testing.

**Results:**

We found a significant relationship between the rs17655 of *ERCC5* gene and the breast cancer risk for the dominant model, OR (95% CI) = 1.24 (1.01–1.52), *p* = 0.036. But we found no significant association between the rs17655 and breast cancer risk for the recessive model (OR = 1.03, 95% CI = 0.80–1.32, *p* = 0.825) and the allele model (OR = 1.16, 95% CI = 0.98–1.38, *p* = 0.083). No statistically significant association were found between the *ERCC5* gene rs17655 and the risk of breast cancer for the dominant model observed in Caucasian populations (pooled OR = 1.03, 95%CI = 0.91–1.18), in African populations (pooled OR = 1.55, 95%CI = 0.77–3.12) and in Asian population (pooled OR = 1.76, 95%CI = 0.755–4.12). No publication bias was observed for three genetic models (*P* = 0.061 for the dominant model, *P* = 0.825 for the recessive model, *P* = 0.118 for the allele model).

**Conclusions:**

Carriers with rs17655 minor allele have higher susceptibility to breast cancer for the dominant model. But no significant relationship was observed between rs17655 and breast cancer susceptibility in Caucasian, African, and Asia populations.

## Introduction

Breast cancer is the most common malignant tumor in female incidence and one of the main causes of female death [1]. According to Global Cancer Statistics 2020 [2], a total of 2.3 million breast cancer patients existed around the world each year, and the incidence rate of breast cancer was increasing year by year. Previously, several risk factors have been reported, such as age, excessive alcohol consumption, excessive fat intake, obesity, alcohol consumption, diabetes, and genetic factors [3–6]. So far, the detailed pathogenesis for the occurrence and development of breast cancer remains unclear. Recently, genetic factors for breast cancer have been extensively investigated, and association between several single nucleotide polymorphisms (SNPs) and breast cancer risk has been reported [7–9].

Previously, several studies [8–12] focused on the correlation between SNPs of the *ERCC5* gene and breast cancer susceptibility, and the SNP-rs17655 (*Asp1104His*) has been mostly studied among many SNPs of *ERCC5* gene. To date, contradictory results also existed among these articles. Although two meta-analyses [9, 13] have been performed, subsequently, several new articles [12, 15–17] on the association between the *ERCC5* gene rs17655 and the risk of breast cancer were published recently. So, we performed this updated meta-analysis to assess the relationship between the *ERCC5* gene rs17655 and the risk of breast cancer.

## Methods

### Publication search

To identify all relevant publications focused on the relationship between *ERCC5* gene rs17655 and the risk of breast cancer, we performed independently and systematically searched related literature from Pubmed, Embase, WanFang database, and China’s National Knowledge Infrastructure. The deadline for literature search and enrollment in eligible studies was January 10, 2025. The following terms for the literature search were used: (“*ERCC5*” OR “excision repair cross-complementing group 5” OR “XPG” OR “rs17655” OR “*Asp1104His*”) AND (“variant” OR “mutation” OR “polymorphism”) AND (“breast tumor” OR “breast cancer”).

### Inclusion and exclusion criteria

Eligible studies were selected when they met the following criteria: (1) focused on the relationship between the *ERCC5* gene rs17655 (*Asp1104His*) and breast cancer susceptibility; (2) sufficient genotype frequencies existed in the article; (3) the selected study should be case-control study; (4) the article should be written in English or Chinese.

Main exclusion criteria: (1) not a case–control study; (2) duplication of previous publications; (3) comment, conference articles, letter, editorial articles, meta- or systematic evaluations; (4) the study could not provide sufficient genotype frequencies. If a study includes multiple ethnic groups, these ethnic groups will be included in the meta-analysis as separate studies.

### Data extraction

Two colleagues extracted data from the enrolled articles independently. The following data were collected, including the family name and abbreviations of given name for the first author, publication years, ethnicity (grouped into Caucasian, African, or Asian), country of the participants, sample size of the case and control group, results for the Hardy-Weinberg equilibrium (HWE). If the same population has published multiple articles, only the one with the largest research sample was eligible. If there were disagreements during the information extraction process, we would resolve them through centralized discussions. If there was still disagreement after the discussion, the third investigator would resolve the disagreement.

### Quality assessment

We also conducted an independent quality assessment for each eligible study according to the Newcastle-Ottawa Scale (NOS) applied to genetic association studies [18]. The NOS assessment methods included three parts: subject selection (0–4), comparability of subjects (0–2), clinical outcome (0–3). The total score ranged from 0 to 9 stars was used for quality assessment, and the total scores above 6 indicate that the article meets the requirement. If different results existed between two evaluators, the inconsistent results would be solved by the third evaluator.

### Statistical analysis

All analysis was performed by using Stata 15.0 version software in this meta-analysis. The pooled odds ratios (ORs) (95%CI) were calculated by the genotype frequencies (Table 1) of every selected study according to standard method, when we performed heterogeneity test for three models, including the dominant, recessive and allele models. The heterogeneity test was performed using the Chi-square-based Q-test (*P* < 0.1 was considered significant) and the I-square test (>50% was considered significant and *P* < 0.10 indicated heterogeneity) [19, 20]. When there was no heterogeneity in the selected studies, the fixed effects model (Mantel-Haenszel) was used to estimate the pooled ORs, and when there was significant heterogeneity among the selected studies, the random effects model (DerSimonian and Laird) was used to estimate the pooled ORs [21, 22]. Sensitivity analysis was also performed to determine the stability of the calculated pooled ORs and 95%CI. Funnel plot methods were used for the test of publication bias using Begg’s linear regression [23, 24].

**Table 1:** Studies and data included in this meta-analysis

**Author**	**Year**	**Ethnicity**	**Sample size**		**Cases**					**Control**					***P*-values for HWE**	**Source of control**	**NOS scores**			
			
			**Case**	**Control**	**Asp/Asp**	**Asp/His**	**His/His**	**Asp**	**His**	**Asp/Asp**	**Asp/His**	**His/His**	**Asp**	**His**			**Subject selection** **0–4**	**Comparability of subjects** **0–2**	**Clinical outcome** **0–3**	**Overall scores** **0–9**
Kumar	2003	Caucasian	220	308	108	96	0.54	PB	128	182	107	19	471	145	0.54	PB	3	2	2	7
Mechanic	2006	Caucasian	1249	1133	771	409	0.69	PB	547	661	412	60	1734	532	0.69	PB	3	2	3	8
Mechanic	2006	African	757	674	231	387	0.51	PB	665	231	320	123	782	566	0.51	PB	3	2	3	8
Shen	2006	Caucasian	154	151	83	63	0.27	Sisters	79	82	62	7	226	76	0.27	Sisters	3	1	3	7
Crew	2007	Caucasian	999	1051	562	371	0.85	PB	503	571	409	71	1551	551	0.85	PB	4	2	2	8
Jorgensen	2007	Caucasian	264	275	159	93	0.78	PB	117	165	95	15	425	125	0.78	PB	4	1	2	7
Rajaraman	2008	Caucasian	819	1079	482	288	0.42	PB	386	674	352	53	1700	458	0.42	PB	4	1	2	7
Smith	2008	Caucasian	320	408	195	113	0.002	HB	137	256	124	28	636	180	0.002	HB	3	1	2	6
Smith	2008	African	52	75	13	32	0.91	HB	46	18	37	20	73	77	0.91	HB	3	1	2	6
Ming-Shiean	2010	Asia	401	531	134	191	0.006	HB	343	159	243	129	561	501	0.006	HB	4	1	2	7
Wang HT	2015	Asia	101	101	95	6	0.802	HB	6	100	1	0	201	1	0.802	HB	3	1	2	6
Ma SH	2016	Asia	320	237	116	145	0.108	HB	263	84	107	46	275	199	0.108	HB	4	1	2	7
Adolf IC	2022	African	263	250	51	121	0.014	PB	303	104	107	39	315	185	0.014	PB	3	1	2	6
Khan I	2023	Asia	430	430	343	80	0.104	PB	94	407	23	0	837	23	0.104	PB	4	1	2	7

PB, population-based; HB, hospital-based; Asp: major allele; His: minor allele

## Results

### Characteristics of studies

**Fig. 1:**
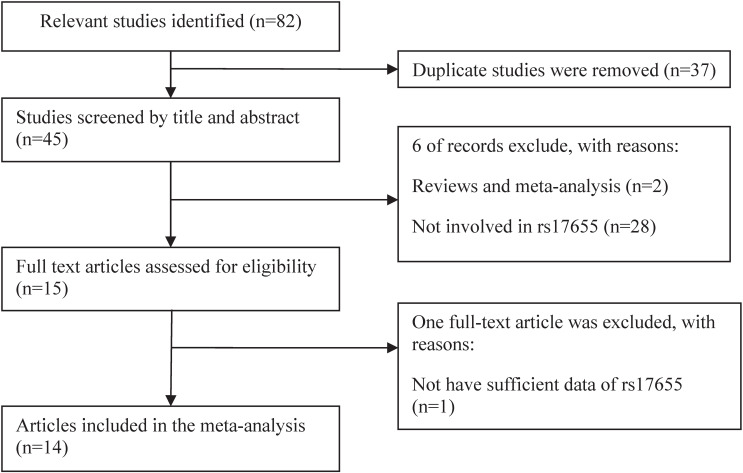
The flow sheet of identification of eligible studies

The flow diagram for the selection of articles was shown in Fig. 1. A total of 82 articles were initially searched, and 37 duplicate publications, 2 reviews and meta-analysis, 28 articles not involved in rs17655 and 1 full-text article that did not have enough genotype data for rs17655 were excluded. We have conducted comprehensive and systematic reading for 45 publications. Finally, 14 studies consisting of 6349 breast cancer patients and 6703 controls were enrolled in the final meta-analysis. Furthermore, the NOS scores of all enrolled studies ranged from 6 to 8 (Table 1).

### Results of the meta-analysis

#### Heterogeneity test

**Fig. 2:**
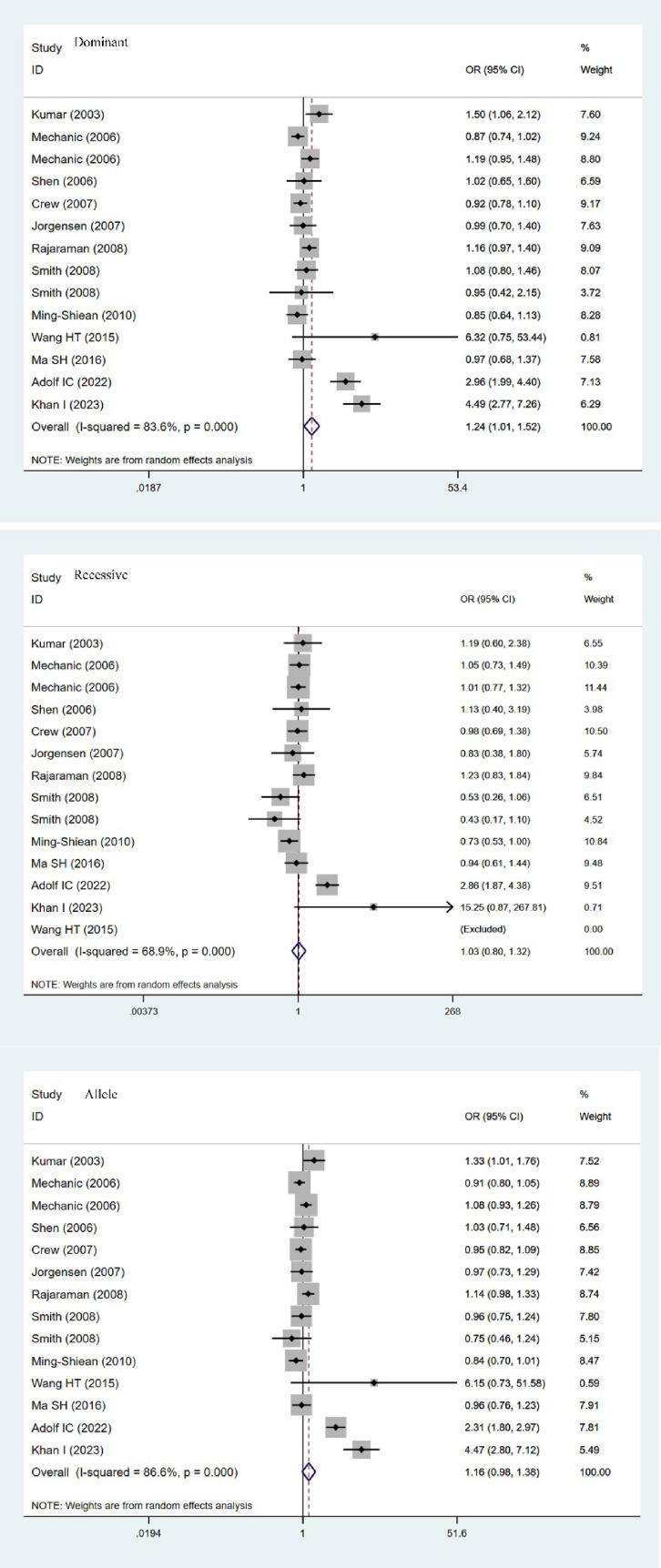
Forest plots of the rs17655 polymorphism for three genetic models (dominant, recessive, and allele models).

**Fig. 3:**
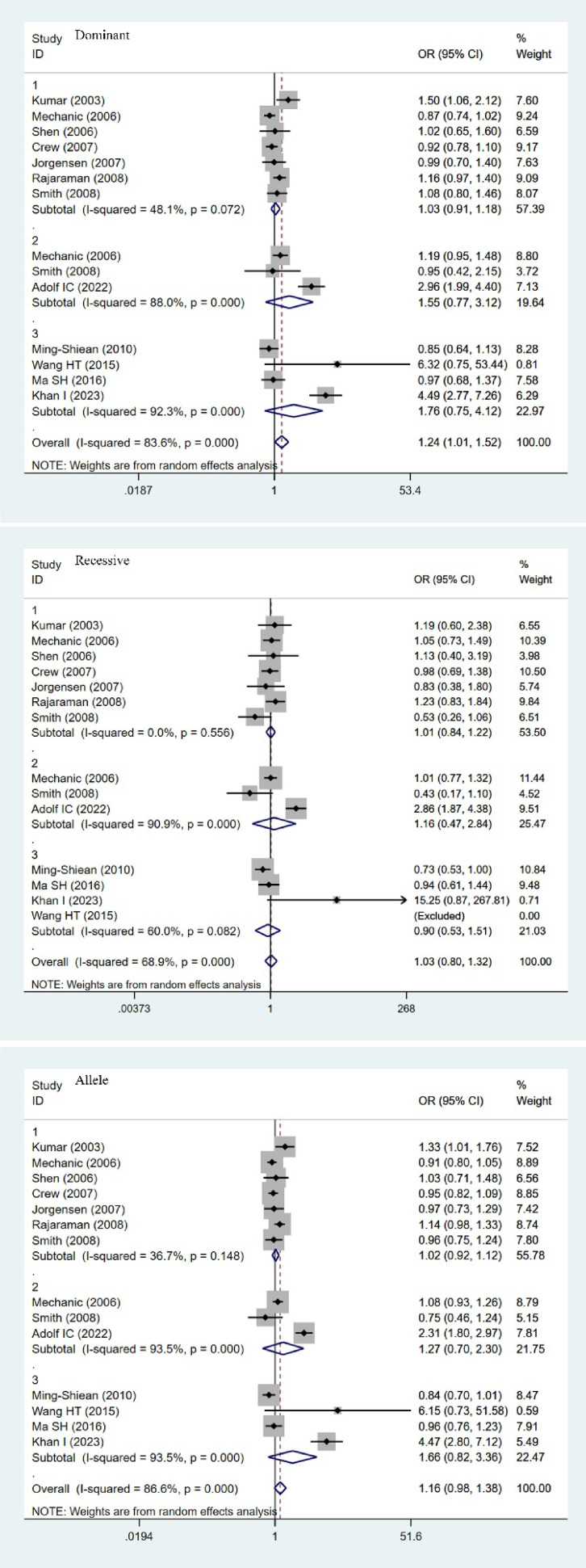
Forest plots of the rs17655 polymorphism for three genetic models grouped by ethnicity. Note: 1: Caucasian; 2: African; 3: Asian

As shown in Fig. 2, we found that there was serious heterogeneity among the selected articles for the dominant model (*I*^2^ = 83.6%, *p* < 0.001), recessive model (*I*^2^ = 68.9%, *p* < 0.001) and allele model (*I*^2^ = 86.6%, *p* < 0.001). Thus, the random-effects model was used for the heterogeneity test and the calculation of pooled ORs. We found a significant relationship between the polymorphisms of the *ERCC5* gene rs17655 and breast cancer risk for the dominant model (OR = 1.24, 95% CI = 1.01 to 1.52, *p* = 0.036), but no significant association was found in the recessive model (OR = 1.03, 95% CI = 0.80 to 1.32, *p* = 0.825) and the allele model (OR = 1.16, 95% CI = 0.98 to 1.38, *p* = 0.083). Subgroup analyzes were also performed according to ethnicity, and the studies were grouped into three groups, including Caucasian, African and Asia (Fig. 3). We did not find any statistically significant association between the *ERCC5* gene rs17655 and the risk of breast cancer for the dominant model observed in three populations (pooled OR = 1.03, 95%CI = 0.91–1.18 for the Caucasian population, pooled OR = 1.55, 95%CI = 0.77–3.12 for the African population and pooled OR = 1.76, 95%CI = 0.755–4.12 for the Asian population). And we did not find a statistical relationship between *ERCC5* gene rs17655 polymorphisms and the risk of breast cancer for recessive and allele models in Caucasian, African, and Asia populations.

#### Sensitivity analysis

**Fig. 4:**
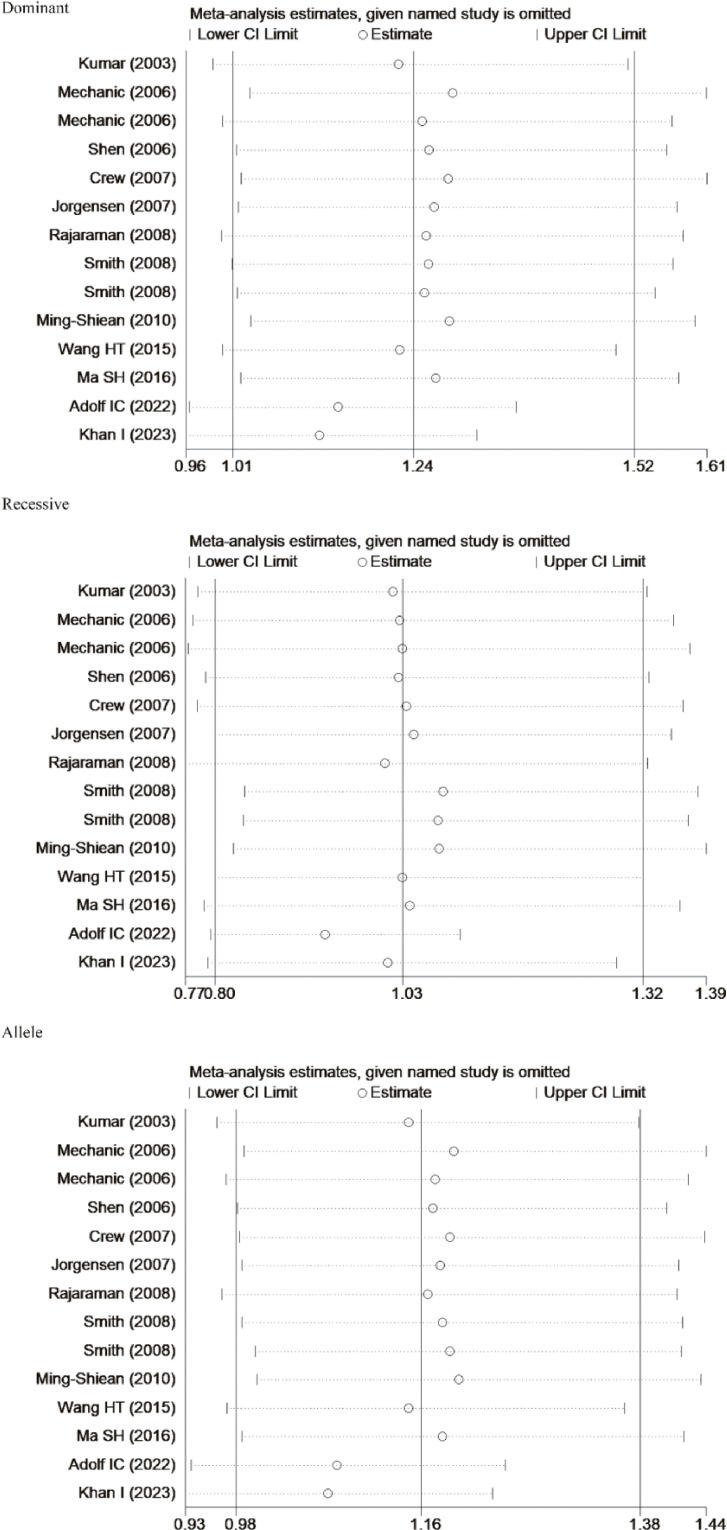
Sensitivity analysis for association between ERCC5- rs17655 and breast cancer risk for three genetic models.

We also performed a sensitivity analysis for all selected studies. As shown in Fig. 4, we found that there was no influence for the pooled ORs when deleted any single study. Therefore, the stability of our research results was good, and the results were reliable.

#### Publication bias

**Fig. 5:**
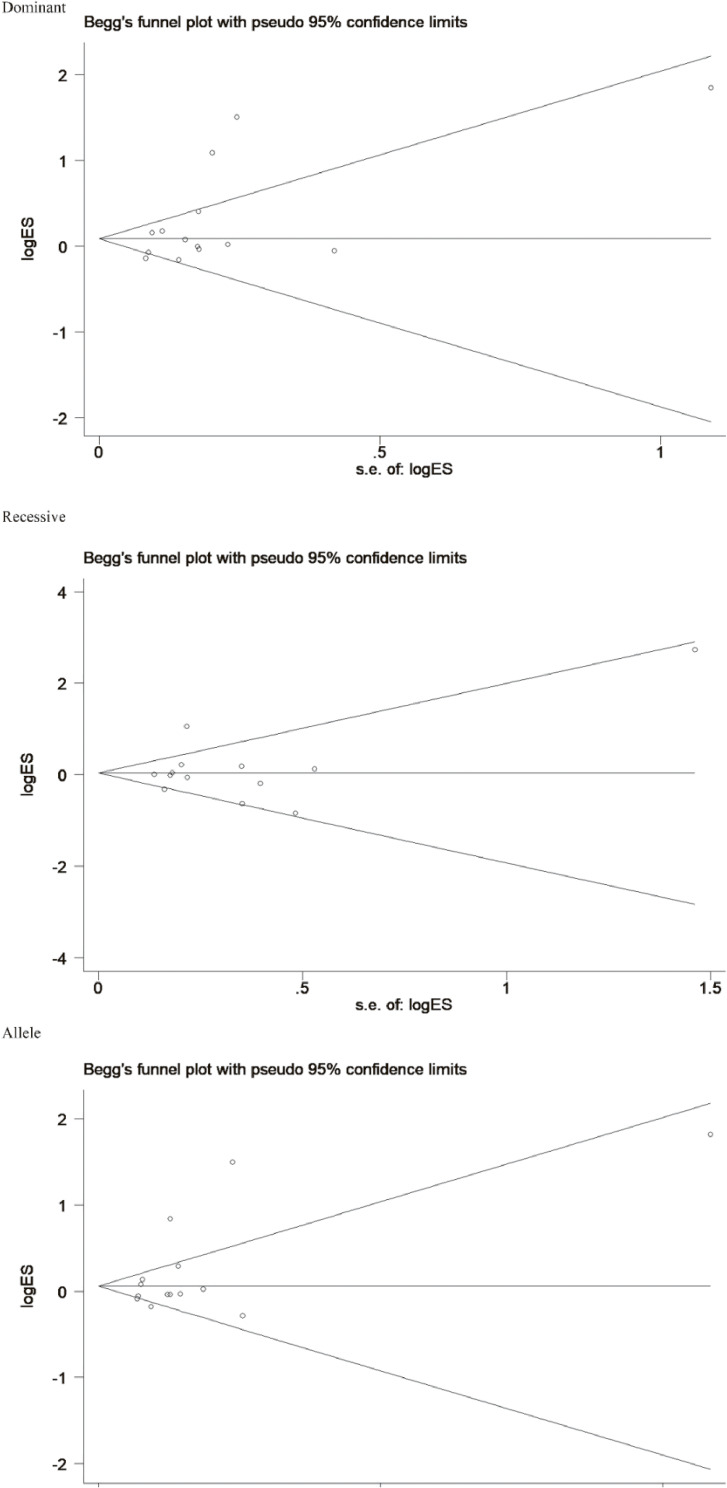
Begg funnel plot for publication bias analysis for three genetic models.

Publication bias testing was also performed using Begg’s funnel plot, the results of which were shown in Fig. 5. No publication bias was observed for three genetic models (*P* = 0.061 for the dominant model, *P* = 0.825 for the recessive model, *P* = 0.118 for the allele model).

## Discussion

In this study, we found that *ERCC5* rs17655 was correlated with an increased risk of breast cancer for the dominant model. Grouped by ethnicity, we did not find a significant relationship between *ERCC5* gene rs17655 polymorphisms and susceptibility to breast cancer for the three genetic models in Caucasian, African and Asian populations. But no statistical relationship was observed between the *ERCC5* gene rs17655 polymorphisms and the risk of breast cancer for recessive and allele models in Caucasian, African, and Asia. Previously, several studies [13–17] were conducted to evaluate the relationship between the *ERCC5* gene polymorphism rs17655 and breast cancer susceptibility; however, to date contradictory results have also been obtained on this topic. Ming-Shiean et al. [14] suggested that the rs17655-G allele (Asp) was significantly associated with increased risk of breast cancer in Asian subjects. Smith et al. [25] suggested that rs17655 minor allele was not associated with breast cancer risk. Mechanic et al. [26] concluded similar results in African Americans and whites. And similar negative results were also obtained from several previous studies [14, 27–30]. Two meta-analyses [9, 13] were previously performed to evaluate the relationship between rs17655 and breast cancer risk. In 2011, Ding et al. [9] conducted a meta-analysis, which included 10 previous articles, indicated that there was no significant relationship between the *ERCC5* gene rs17655 polymorphism and susceptibility to breast cancer. In 2014, another meta-analysis [13] concluded similar results. The fore-mentioned two meta-analyses did not group the selected articles by ethnicity; furthermore, subsequently four articles [12, 15–17] were newly published recently. Recently, Khan [11] indicated that there was no association between *ERCC5* rs17655 and breast cancer risk in Pakistan. Wang et al. [12] did not find a significant relationship between rs17655 polymorphisms and the risk of breast cancer. Ma et al. [16] also suggested that rs17655 does not influence the development of breast cancer in a Chinese population. Kumar et al. [31] found that the rs17655- C allele (His) was significantly associated with an increased risk of breast cancer in Europeans. And another recently published study [17] suggested that *ERCC5*- rs17655 has both independent and interactive effects on susceptibility to breast cancer in Tanzanian women. The different results obtained from these studies may be due to several factors, such as ethnicity, different sources of control, and sample size. And in this meta-analysis, we concluded different results with fore-mentioned two meta-analyses, we also grouped the studies into three groups according to ethnicity, and different results were also obtained among three ethnic groups.

Genomic instability caused by the loss of expression of DNA damage repair related genes and the inactivation of some expression products was one of the important reasons for the occurrence of breast cancer [32]. The nucleotide excision repair (NER) system plays an important role in DNA repair. The *ERCC5* gene was an important component of the NER system. The basic structure of human ERCC5 protein includes highly conserved N- and I-nuclease domains, which together form the nuclease core. The N- and I-nuclease domains were separated by 600 amino acids that constitute a critical region for protein-protein interactions, including with transcription factor IIH (TFIIH) and replication protein A (RPA) and combine ERCC5 with the sites of NER [33, 34]. *Asp1104His* (G>C) polymorphism (rs17655) can lead to the transformation of aspartic acid at position 1104 of exon 15 to histidine, thereby affecting protein activity and interaction with TFIIH, affecting the NER system and altering the cancer susceptibility [35, 36].

There were also several limitations in this study. Firstly, only articles written in English or Chinese were searched and data of articles written in other languages was lost. However, publication bias was tested in the study, and the results suggested no publication bias existed in this study. Secondly, among the 14 selected articles, the genotype distributions in 2 articles were not consistent with HWE, which may influence the stability of the results. Thirdly, there was no unified control group source for these studies included in the analysis. The source of control group includes community population, hospital population and sisters. However, sensitivity analysis has indicated that sufficiently shows that these studies did not significantly influence the overall results. Lastly, the sample size of this meta-analysis was relatively small, so more case-control studies were needed in the future, and the results obtained in this study should be verified by future meta-analysis with larger sample size.

In conclusion, we found that the minor allele rs17655 correlated with an increased risk of breast cancer for the dominant model. But no significant relationship was observed between rs17655 and breast cancer susceptibility in Caucasian, African, and Asia populations. In the future, more studies should be performed to verify the relationship between rs17655 and breast cancer susceptibility in different populations.

## Abbreviations

CNKI: China National Knowledge Infrastructure
ERCC5: Cross-complementation group 5
NOS: Newcastle-Ottawa Scale
OR: odds ratio
CI: confidence interval
HWE: Hardy-Weinberg equilibrium

## Declarations

### Data availability statement

The data and figures generated and analyzed during this study are available from the corresponding authors upon reasonable request.
